# The Difference between PC-Based and Immersive Virtual Reality Food Purchase Environments on Useability, Presence, and Physiological Responses

**DOI:** 10.3390/foods13020264

**Published:** 2024-01-15

**Authors:** Shelley Woodall, James H. Hollis

**Affiliations:** Department of Food Science and Human Nutrition, Iowa State University, Ames, IA 50312, USA; swoodall@iastate.edu

**Keywords:** consumer behavior, virtual reality, presence, fast food restaurant, supermarket

## Abstract

Computer simulations used to study food purchasing behavior can be separated into low immersion virtual environments (LIVE), which use personal computers and standard monitors to display a scene, and high immersion virtual environments (HIVE) which use virtual reality technology such as head-mounted displays to display a scene. These methods may differ in their ability to create feelings of presence or cybersickness that would influence the usefulness of these approaches. In this present study, thirty-one adults experienced a virtual supermarket or fast-food restaurant using a LIVE system or a HIVE system. Feelings of presence and cybersickness were measured using questionnaires or physiological responses (heart rate and electrodermal activity). The participants were also asked to rate their ability to complete the set task. The results of this study indicate that participants reported a higher sense of presence in the HIVE scenes as compared to the LIVE scenes (*p* < 0.05). The participant’s heart rate and electrodermal activity were significantly higher in the HIVE scene treatment when compared to the LIVE scene (*p* < 0.05). There was no difference in the participant’s ability to complete tasks in the different scenes. In addition, feelings of cybersickness were not different between the HIVE and LIVE scenes.

## 1. Introduction

A poor diet is linked to several chronic diseases, including cardiovascular disease [1], some types of cancer [2], type 2 diabetes [3], and obesity [4]. It has been estimated that 11 million premature deaths and 255 million disability-adjusted life years are attributable to dietary risk factors [5]. In addition, our food choices have a significant impact on the environment [6]. Studies indicate that changing our dietary choices would improve health and reduce environmental degradation [7]. Although dietary habits are often thought to be difficult to change, diets are in a state of constant flux and can change markedly within a generation [8]. In a typical week, 87% of American households purchase food from a grocery store or supermarket, and 85% acquire food from restaurants [9]. Consequently, changing purchasing habits at these locations may help improve health and reduce the impact of diet on the environment. However, identifying strategies that align dietary choices with societal goals while identifying unintended consequences, such as exacerbating existing nutritional inequalities, would be facilitated by the development of new methods to understand consumer purchasing behavior.

Several experimental approaches can be used to understand the effect of the environment on food choices. These include focus groups [10,11], laboratory studies [12], studies that observe consumers in real-life food outlets [13], and studies that use test food outlets or studies using physical simulations of food outlets [14,15]. Each of these methods has strengths and weaknesses. Focus groups provide insight into what consumers are thinking, but it is not clear that their stated food choices actually reflect their food choices in real-life settings [16]. Laboratory studies offer strong experimental control but do not reflect the environment in which food choices are made, and data from these studies may not accurately predict behavior in real-life settings as several studies report that the environment can influence food purchase decisions [17,18,19]. Field studies that observe consumers in real-life food outlets are the gold-standard for studying food choices, as they observe shoppers who are exposed to the full range of environmental factors that may influence their behavior. Crucially, the shoppers’ actions are not “zero-stakes” and have real consequences (e.g., they must spend their own money and eat the food they purchase). These studies can also highlight important antecedents to a food purchase (e.g., route taken through the store, time viewing objects, number of foods lifted), which may provide insights into how food purchase decisions are made. However, there are substantial logistical barriers to conducting field studies, as many retailers may be reluctant to allow researchers into stores or restaurants to conduct research. The ability to change pricing, store layout, or shelf placement may also be limited [20]. Other drawbacks to field studies include the cost, the time required to collect data, limited experimental control, difficulties collecting physiological data that may provide insights into purchasing behavior, and difficulties independently replicating this study [21]. An alternative to field studies is to create physical replications of food outlets to investigate food choices [22]. While this, to some extent, would replicate the context in which food choices are made and allow for changes to the food environment, the creation of physical replicas of food outlets requires substantial resources, including space, and may only be available to a small number of researchers, limiting research in this area. Due to the weaknesses of focus groups or laboratory studies and the logistical, cost, and time issues with conducting field studies or testing food outlets/physical simulations of food outlets, the development of alternative approaches to study food choices is required to facilitate innovative approaches to promote food choices that improve health and reduce the environmental impact of the diet. Virtual simulations of grocery stores or restaurants may provide a useful approach to understanding consumer behavior or to refine interventions before they are implemented in real-life settings.

Computer simulations using 3D computer graphics to replicate food outlets are an emerging approach to studying food purchasing behavior. These simulations can be experienced using video walls [22,23], PC Monitors [24,25], immersive Virtual Reality (VR) headsets [26,27], CAVE virtual reality systems [28], or augmented reality [29,30]. These approaches can be broadly split into high-immersion virtual environments (HIVE) and low-immersion virtual environments (LIVE). The use of HIVE is particularly intriguing, as VR head-mounted displays (VR-HMD) have become relatively inexpensive and have the potential to create a sense of presence. The sense of presence causes the user to suspend disbelief and believe they are actually in the virtual environment, physically and emotionally reacting to stimuli created by the computer-generated application as if they were in the real world [31]. This may be an important benefit when studying food purchasing habits, as studies show that the environment [32,33,34,35] and emotions [36,37] can influence behavior. Consequently, food purchasing behavior in HIVE may more accurately reflect real-life behavior than simulations experienced using LIVE. 

The sense of presence can be measured using questionnaires [38] or physiological markers such as heart rate [39], electrodermal activity (EDA), or electroencephalograms [40]. A previous study that used a questionnaire found that participants who experienced a HIVE supermarket experienced a greater sense of presence than a LIVE version [26]. While questionnaires are a common method to measure presence, they may be subject to response biases and yield inaccurate information [41]. Consequently, physiological markers of presence may yield additional evidence regarding feelings of presence in a virtual environment. To date, studies have investigated the effects of food cues experienced using VR on physiological measures such as heart rate, skin conductance [42], or salivation [43,44], but further research is required to determine how individuals respond in virtual food environments. 

While HIVE can create a sense of presence, it can also elicit feelings of cybersickness [45]. It is believed that cybersickness is due to a perceptual conflict between the visual system (which reports that the user is moving) and the vestibular system (which reports that the user is stationary) [46]. The symptoms of cybersickness are not trivial and include nausea, pale skin, cold sweats, vomiting, dizziness, headache, dryness of mouth, disorientation, and fatigue [47]. It has been found that up to 80% of immersive VR users experience some cybersickness [48,49], although current-generation virtual reality head-mounted displays may significantly reduce feelings of cybersickness [50]. While most users recover within an hour, some effects can last for several hours [45]. This has implications for HIVE technology. If participants experience cybersickness, it may influence their "food choices" in a virtual environment. Moreover, it may affect their ability to finish a test session, or they may not return for further test sessions [51].

For virtual environments to be useful, they must be usable by the target study population, and users should find the HIVE methods to be equally as usable as the LIVE methods. A potentially key aspect of usability is how users navigate through the virtual store. In LIVE scenes, a keyboard or joystick can be used to navigate through the scene. In HIVE scenes, there are multiple methods to navigate the scene, including the use of the thumbstick on handheld controllers or using the controllers to ‘teleport’ (i.e., the user points a laser pointer at the spot they want to move to and presses the controller trigger, and they automatically appear in the new position). The differences in how people move through the store may lead to differences in the products that users view (teleporting may mean that users miss products on the shelves that they ‘skip’ by). In addition, if interaction with the application menus to ‘purchase’ foods or obtain information about foods is not intuitive or awkward, the user may choose fewer foods than they would normally select in order to complete this study faster. Again, multiple options are possible, and participants can interact with the menus using a mouse (LIVE scenes) or through a ‘laser pointer’ (HIVE scenes). In this present study, voice recognition technology was used to interact with menus to investigate another potential option to interact with the application features.

The objective of this study was to determine differences in presence (measured using a questionnaire, heart rate, and EDA), cybersickness (using a questionnaire), and the participant’s subjective assessment that they could accomplish a set task (using a questionnaire) when experiencing a LIVE or HIVE supermarket or restaurant. It is hypothesized that there will be increased feelings of subjective presence, heart rate, EDA, and cybersickness when experiencing the HIVE scenes. We also hypothesize that there will be no difference in usability between the LIVE and HIVE scenes. 

## 2. Methods

### 2.1. Participants

Individuals were informed about this study through an email sent to all faculty, students, and staff at Iowa State University or through word of mouth in the local Ames, Iowa, community. Potential participants were informed about this study and, if they remained interested in participating, were asked to sign an informed consent form. After signing the informed consent form, the participant completed a screening questionnaire to confirm their eligibility for this study. If the participant was eligible for this study, they were randomized to a treatment order. Thirty-one participants were recruited subject to the following inclusion criteria: age between 18 and 60 years. Potential participants were excluded if they have a history of motion sickness, experience seizures of any type, have been diagnosed with a seizure disorder, or have an allergy to adhesives. This study was conducted according to the guidelines laid down in the Declaration of Helsinki, and all procedures involving human subjects/patients were approved by the Institutional Review Board (IRB) at Iowa State University (IRB ID number 19-166, date of Approval–13 May 2019). Written informed consent was obtained from all subjects/patients. 

### 2.2. Virtual Worlds

For this study, four computer-generated, three-dimensional (3D) scenes were developed. These were: HIVE supermarket (HIVESM), LIVE supermarket (LIVESM), HIVE fast-food restaurant (HIVEFFR), and LIVE fast-food restaurant (LIVEFFR). The scenes were all identical except for the level of immersion and the method used to navigate around the scene. The virtual scenes were created using the Unity game engine (version 2018.4, Unity Technologies, San Francisco, CA, USA). The 3D models used to create the scenes were purchased from Turbosquid (www.turbosquid.com, accessed on 3 December 2023) or the Unity Asset Store (www.assetstore.com, accessed on 3 December 2023).

**Figure 1:**
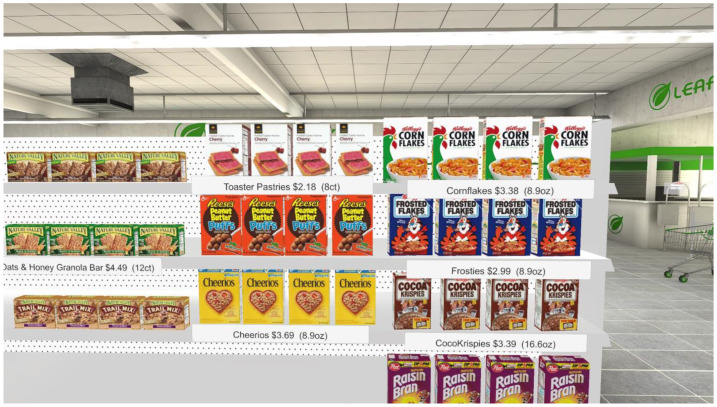

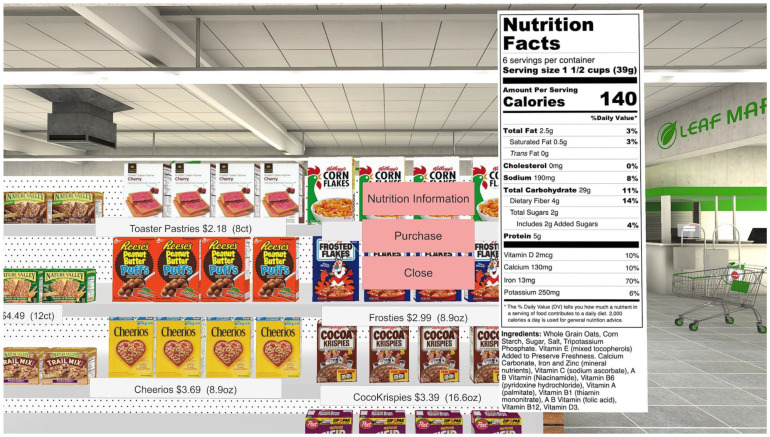
The virtual supermarket. A menu for selected foods could be selected that allowed the participant to obtain nutrition information about the product.

The supermarket scene simulated a medium-sized, modern supermarket with models of many foods that are commonly available in a United States supermarket (Figure 1). The participants could obtain nutrition information about food products or purchase foods using voice commands. When participants said the name of a product, a menu appeared in front of that product. When this menu was open, if they said "nutrition,” a nutrition information panel would appear. If they said "purchase,” the item was purchased. The participant heard background sounds of a busy supermarket, including background conversations, announcements made over the supermarket intercom, and the sound of cash registers being operated. Voice recognition used the inbuilt voice recognition features of the Unity game engine.

**Figure 2:**
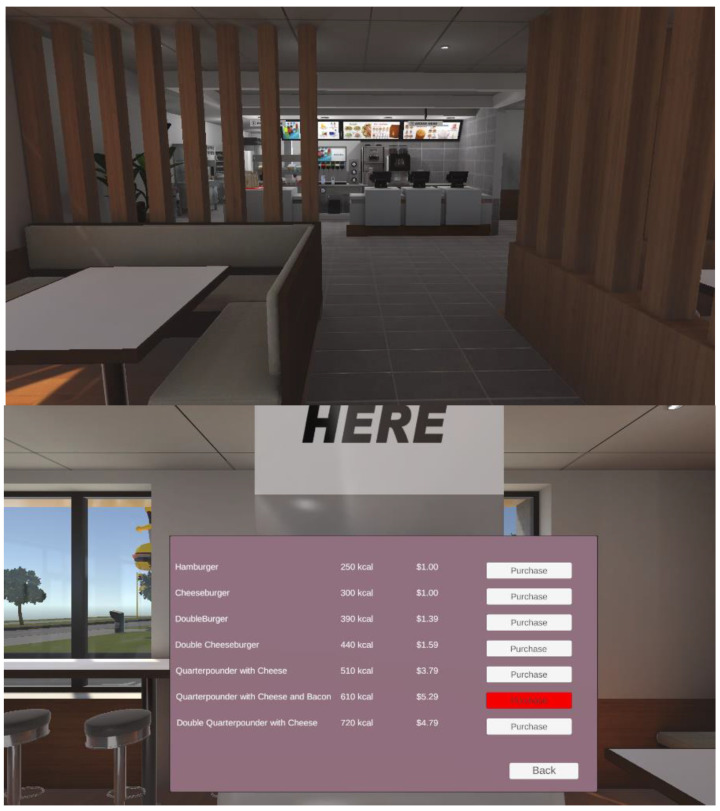
The virtual fast-food restaurant. A menu for selected foods could be selected that allowed the participant to obtain nutrition information about the product.

The restaurant scene simulated a modern-fast food restaurant (Figure 2). The participant interacted with menus on a terminal to select and purchase foods. Similar to the supermarket treatments, the participants used voice commands to select and purchase foods. However, no nutrition information was provided other than calorie information. Background sounds of a busy restaurant were added, which included background conversations, restaurant equipment being operated, and orders being taken.

For the HIVESM and HIVEFFR, the participant could move around the virtual worlds using the hand-held wands that accompany the HTC Vive. The participant moved via ‘teleportation’. In this method, when the participant places their finger on the trackpad, it emits a laser beam from a graphical representation of the wand in the VR space. To move, the participant points to the place they want to move to and then presses the trackpad button to move there. For the LIVESMPC and LIVEFFR, participants navigated throughout the store or restaurant using a first-person avatar that was controlled using a Logitech Extreme 3D Pro joystick (Logitech, CA, USA). Moving the joystick forward/back/left/right would move the avatar in that direction. A ‘top-hat’ joystick (situated on top of the main joystick) was used to simulate head movement so different aspects of the store or restaurant could be viewed. However, this movement was constrained to 90° to the left and right or up and down so that the participant could not rotate the ‘head’ through a full 360° range of motion. 

A PC (Dell Computers) with the following specifications was used for all aspects of this study: an Intel i7 processor, 16 GB of RAM, an Nvidia GTX1070 graphics card, and Logitech Z200 Stereo speakers (Logitec, CA, USA) were used to produce the restaurant or supermarket sounds. For the PC scenes, participants viewed the scenes on a 21-inch Dell monitor that had a resolution of 1024 × 800. For the VR treatments, the same scenes were experienced while wearing an HTC Vive head-mounted display (VR-HMD; HTC, Taoyuan City, Taiwan). 

### 2.3. Questionnaires

At the beginning of the first test session, participants completed a questionnaire that collected demographics, educational background, food purchasing habits, attitudes toward food, understanding of computer technology, experience with playing computer games, familiarity with virtual reality, and confidence in navigating computer simulation information. Immediately after leaving the simulation, participants completed the Slater-Usoh-Steed (SUS) presence questionnaire, which captures responses on a 7-point Likert scale [52]. The participant was also asked to rate their feelings of cybersickness and how well they thought they accomplished the task given to them on a 7-point Likert scale. The questionnaires were administered using Qualtrics software (Qualtrics 06,2019Version adde, Provo, UT, USA), and responses were collected using a personal computer. 

### 2.4. Physiological Measures

The skin area was cleansed with an alcohol swab before surface electrodes were attached to the right forearm, right index finger, right middle finger, and left and right inner ankles to capture heart rate and EDA data. Medical-grade tape was used to ensure the electrodes were secure throughout the testing session. The surface electrodes were connected to a Biopac MP36R (BIOPAC Systems Inc., Goleta, CA, USA). AcqKnowledge (v5.0) software (BIOPAC Systems Inc., Goleta, CA, USA) were used to extract data features. 

### 2.5. Procedure

Participants reported to the laboratory at a time that was convenient to them between 10 a.m. and 4 p.m. They were required to report to the laboratory at the same time for each of the test sessions and were asked not to eat for at least two hours before each test session. First, the surface electrodes were attached, and the participant was asked to sit quietly for ten minutes so that baseline physiological measurements could be collected. Then, for the HIVE treatments, the VR headset was placed on the participant’s head, and the relevant scene was shown. For the LIVE treatments, the relevant scene was shown on the PC monitor. The participant was provided with full instructions about movement through the scene and the voice commands used to interact with menus. While in the restaurant scene, the participant was asked to use the menus to select food items and ‘purchase’ the chosen item. They were asked to ‘purchase’ a meal containing a sandwich, a side, and a beverage. Then, they were asked to move around the restaurant for at least five minutes. When exploring the supermarket’s aisles, the participant was asked to locate the cereal and bread sections and use the menu selections to read the nutritional information of two specific products (white bread and Cheerios cereal). A researcher was present to confirm the participant accomplished these tasks by viewing the participant’s actions on a PC monitor. The nutrition information presented were based on USDA (United States Department of Agriculture) data, and prices reflected local food outlet values (at the time of this study) when creating the programs. Nutrition facts labels were constructed following the current United States FDA (Food and Drug Administration) labeling guidelines. At the end of viewing the supermarket or restaurant scene, the VR HMD (in the VR scenes) was removed, and the participant completed the SUS, cybersickness, and usability questionnaires. 

### 2.6. Statistical Analysis

Means and standard error of means were calculated for all participant responses and study variables. Differences between treatments were determined using a repeated measure ANOVA, with the condition as a fixed effect variable and the participant as a random effect variable. Post hoc analysis was conducted using Tukey’s honest significance difference (HSD) test. Statistical significance was set at *p* < 0.05 to determine the effect of the condition on response. All statistical analyses were completed using JMP Pro 15.0 software (SAS, Cary, NC, USA). 

## 3. Result

### 3.1. Demographics

This study group was predominantly female (68% female/32% male) and was in the 18–25 year age group. Participants had a self-reported body mass index of 24.0 (SD = 4.2, range 18.5 to 37.8). Most had some college experience or a 4-year degree and used a computer daily (52%). However, the majority (77%) “never” played computer games, and 58% had not experienced VR before their participation in this study. Most participants visited restaurants (52%) and did their grocery shopping (84%) “once per week.” Almost half (48%) of participants reported they were “always” responsible for buying groceries in their household.

### 3.2. Questionnaires

Each of the questions from the presence questionnaire was analyzed individually (Table 1). For the question “rate your sense of being in the SM/FFR scene,” there was a statistically significant effect of condition on response (f(3,90) = 37.8, *p* < 0.0001). Post hoc analysis indicated that the participants had a greater sense of being in the HIVE supermarket/fast food restaurant (*p* < 0.05). For the question “to what extent were there times during the experience when the SM/FFR was the reality for you?” There was a statistically significant effect of condition on response (f(3,90) = 44.5, *p* < 0.0001). Post hoc analysis indicated that the participants had a greater sense of feeling that the supermarket/fast food restaurant were the reality in the HIVE condition (*p* < 0.05). For the question “Was the SM/FFR more like images that you saw OR more like somewhere that you visited?” there was a statistically significant effect of condition on response (f(3,90) = 18.9, *p* < 0.0001). Post hoc analysis indicated that the participants had a greater sense of somewhere that they visited in the HIVE conditions (*p* < 0.05). For the question “Which was strongest, your sense of being in the SM/FFR or of being elsewhere?” There was a statistically significant effect of condition on response (f(3,90) = 23.4, *p* < 0.0001). Post hoc analysis indicated that the participants had a greater sense of being in the supermarket/fast food restaurant when in the HIVE condition (*p* < 0.05). For the question “I think of the SM/FFR as a place in a way similar to other places that I’ve been today,” There was a statistically significant effect of condition on response (f(3,90) = 8.7, *p* < 0.0001). Post hoc analysis indicated that the participants had a greater sense of feeling like they had been in a similar place today when in the HIVE condition (*p* < 0.05). For the question “Did you often think to yourself that you were actually in the SM/FFR?” There was a statistically significant effect of condition on response (f(3,90) = 17.6, *p* < 0.0001). Post hoc analysis indicated that the participants had a greater sense of thinking they were in a supermarket/fast food restaurant when in the HIVE condition (*p* < 0.05). For the question “Rate the extent to which you were aware of background sounds in the laboratory where this was actually taking place,” there was no statistically significant effect of condition on response (f(3,90) = 2.6, *p* = 0.058). For the question “How dizzy, sick, or nauseous did you feel during or as a result of the experience?” There was no statistical difference between conditions, and only minimal cybersickness was reported after participating in each treatment session (*p* > 0.05). All participants successfully completed the tasks, and for the question “Overall, how well do you think that you achieved your task?” There was no statistical difference between treatments (*p* > 0.05). 

### 3.3. Physiological Measurements

Table 2 provides data regarding the physiological measurements. There was a statistically significant effect of condition on change in heart rate (f(3,90) = 21.4, *p* < 0.0001). Post hoc analysis indicated that the participants heart rate was higher when they were in the HIVE scenes (*p* < 0.05). There was also a statistically significant effect of condition on change in electrodermal activity (f(3,90) = 5.1076, *p* < 0.0023). Post hoc analysis indicated that the participants’ EDA increased when they were in the HIVE scenes (*p* < 0.05).

### 3.4. Time in the Scenes

Participants spent 5.8 min (SEM = 0.2) in the LIVEFFR scene, 6.6 min (SEM = 0.3) in the HIVEFFR scene, 6.0 min (SEM = 0.23) in the LIVESM scene, and 7.0 min in the HIVESM scene. There was a statistically significant effect of condition on time spent in the scenes (f(3,90) = 6.8237, *p* = 0.003). Post hoc analysis found that individuals spent longer in the HIVESM scene than in the LIVESM and LIVEFFR scenes (*p* < 0.05).

## 4. Discussion

In this present study, we hypothesized that there would be increased feelings of subjective presence, heart rate, EDA, and cybersickness when experiencing the HIVE scenes. We also hypothesized that there would be no difference in usability between the LIVE and HIVE scenes. The HIVE scenes did increase feelings of subjective presence, heart rate, and EDA, and this hypothesis was accepted. However, participants did not report increased cybersickness when in the HIVE scenes, and this hypothesis was rejected. We did not find any differences in participants’ ratings of the usability of the scenes. These data add to the growing literature suggesting that virtual environments may be a useful approach to understanding food purchasing behavior.

In this present study, the participants were predominantly young, educated, had little experience with VR, and did not regularly play video games. However, most use computers in their daily lives. This study group was relatively homogenous and limited in size and did not allow for questions about previous experience with VR or computer games, age, or gender on the outcome measures to be investigated. It is possible that age will have an effect on a person’s experience using VR. For instance, younger people likely spend larger amounts of time in virtual worlds, which may change their feelings of presence when in a virtual supermarket or restaurant. In addition, their familiarity with virtual worlds may help them navigate through the virtual worlds using the user interface.

The HIVE applications created a greater sense of subjective presence among the users. When designing effective VR scenes, it is essential to create a sense of presence so that users suspend disbelief, believe they are actually present in the VR environment, and respond as they would in equivalent real-life situations [53,54]. While there is no generally accepted measure of ‘presence’, it has been proposed that questionnaires are the preferred method [39]. However, there are several issues with using questionnaires to determine presence. First, participants may respond to questions in idiosyncratic ways. In one study, participants were asked the question, “Please rate your sense of being in the office space." Participants in a real office space only rated their sense of being in the office as four on a seven-point scale [52]. Presumably, the participants recognized they were inhabiting reality but were possibly comparing the office to their model of what an office should look like, and the low score reflected the discrepancy. Relevant to this present study, it has been suggested that using questionnaires across different types of environments (e.g., immersive VR v desktop PC) has limited utility [52], and these data should be interpreted cautiously.

A major limitation when using questionnaires is that participants may guess the purpose of this study, especially as it may be difficult to blind the participants or researchers and provide responses to questions that they believe the researchers are looking for (demand bias). Physiological markers of presence that provide an objective measure of presence may overcome this limitation. In this present study, heart rate and EDA were measured, with participants exhibiting higher measures in the VR scenes. It is likely that the most useful physiological markers of presence in studies of restaurants mirror the physiological responses observed when an individual is in a real-life restaurant or supermarket [53]. These may include measures of arousal such as heart rate, heart rate variability, or electrodermal activity [53]. In addition, the effect of HIVE on endocrine and metabolic markers may also be useful. When exposed to food cues, there are a number of physiological responses collectively termed the cephalic phase response (CPR). Studies suggest that CPR is related to the metabolic response to foods or meal size [55,56,57]. Little is currently known about how the environment influences CPR, and IVR may provide an approach to studying this phenomenon. 

The future development of HIVE food outlets should focus on determining the factors that increase the sense of presence. Primarily, the use of equipment that promotes presence by having high resolution and a good field of view is important [58,59]. Improving the fidelity of food models, menus, interactions, odors, sounds, and haptic feedback would likely increase the realism of the experience and elicit behaviors that better reflect real-life situations. However, it is not clear that increasing realism will increase presence in all situations [59]. In this present study, several participants provided anecdotal reports that in the HIVE supermarket treatment, they felt cold when moving through the freezer section of the store. Moreover, they noticed incongruences between the HIVE supermarket/restaurant and real-life that did not meet their expectations (e.g., the lack of soap by the sink in the fast-food restaurant kitchen). They did not mention these after being in the LIVE simulations. Care should be taken when designing HIVE environments so that they match participants’ experiences of real-life settings, as incongruences may reduce their presence. 

In the present study, participants in the IVR scenes did not report feelings of cybersickness. However, participants spent an average of 7.0 min in the SMVR scene and 6.6 min in the FFRVR. One study found that 61% of users experienced cybersickness during a 20 min exposure to IVR [60]. Most symptoms were reported towards the end of the 20 min period. Consequently, this study may have been of insufficient duration to elicit feelings of cybersickness. In HIVE restaurants, the exposure time may be short as it is relatively quick to order a meal. However, for complex tasks in a HIVE supermarket (e.g., buying a week worth of groceries), it may take 20–30 min. The ability of individuals with different demographic characteristics to remain in HIVE for up to 30 min to complete complex tasks requires investigation. In a previous study of HIVE, between 4 and 16% of people terminated their participation before the allotted time was over [61]. If these results hold in HIVE supermarkets, then this would seriously curtail the usefulness of IVR supermarkets and potentially restrict it to more focused questions.

The method of locomotion may reduce feelings of cybersickness. Bodytrackers can be used so that when a participant walks in ‘real-life’ they move in the virtual scene. This may reduce the congruency between the information that the visual system is receiving and the information that the vestibular system is receiving, reducing feelings of cybersickness. However, this approach may require substantial amounts of space, depending on the size of the scene. Participants may also feel uncomfortable walking around a space while wearing a head-mounted display. Alternative methods include using a handheld controller to move through the scene (e.g., the Oculus Rift handheld controller or a gamepad) or teleportation, where the participant points using a controller to a point they want to move to and then presses a button to instantly move there. These methods require less space than using bodytrackers that measure the movement of someone walking in a room. Teleportation may cause lower sensations of cybersickness than steering methods [62]. 

Participants were able to complete the tasks asked of them and self-reported that they completed the tasks adequately in each of the treatments. It is crucial that applications are usable for a wide range of individuals, and further research is required to determine whether these applications can be used adequately by a wider cross-section of society. In particular, individuals who do not commonly use computers, play video games, or have been exposed to virtual reality.

## 5. Limitations

It is important to note that this study has several limitations. First, this study is exploratory in nature and uses a relatively small sample size. Consequently, results from this study require confirmation by larger studies. Second, further studies are required to determine the effect of age, experience with computers or IVR, educational background, presence (measured using questionnaires and physiological markers), cybersickness, and usability in virtual supermarkets/restaurants. Third, the participants spent a relatively short amount of time in IVR. Further research is required to determine if cybersickness symptoms appear after longer periods of time in IVR or if tiredness increases. Fourth, the effect of repeated exposure to IVR on presence and cybersickness requires further investigation (e.g., is the sense of presence or cybersickness reduced with repeated exposure?).

## Figures and Tables

**Table 1:** Participant responses to questions related to sense of presence following participation in each treatment session. A significantly higher sense of presence is reflected in the VR scenes as compared to the traditional PC monitor. N = 31 for all scenes. Results are Mean (SEM). Results with a different superscript are significantly different (*p* < 0.05).

	LIVESM	HIVESM	LIVEFFR	HIVEFFR	F Ratio	*p*-Value
Rate your sense of being in the SM/FFR scene.	3.4 (0.3) ^a^	5.8 (0.2) ^b^	2.9 (0.3) ^a^	6.0 (0.2) ^b^	f(3,90) = 37.8	*p* < 0.0001
To what extent were there times during the experience when the SM/FFR was the reality for you?	2.1 (0.3) ^a^	4.6 (0.3) ^b^	2.1 (0.3) ^a^	5.2 (0.3) ^b^	f(3,90) = 44.5	*p* < 0.0001
Was the SM/FFR more like images that you saw OR more like somewhere that you visited?	2.4 (0.3) ^a^	4.7 (0.3) ^b^	2.7 (0.4) ^a^	5.0 (0.3) ^b^	f(3,90) = 18.9	*p* < 0.0001
Which was stronger, your sense of being in the SM/FFR or of being elsewhere?	2.8 (0.3) ^a^	5.2 (0.2) ^b^	2.9 (0.4) ^a^	5.4 (0.2) ^b^	f(3,90) = 23.4	*p* < 0.0001
I think of the SM/FFR as a place similar to other places that I’ve been today.	3.2 (0.3) ^a^	4.7 (0.3) ^b^	3.7 (0.4) ^a^	5.0 (0.3) ^b^	f(3,90) = 8.7	*p* < 0.0001
Did you often think to yourself that you were actually in the SM/FFR?	2.2 (0.3) ^a^	4.0 (0.4) ^b^	2.0 (0.3) ^a^	4.7 (0.3) ^b^	f(3,90) = 17.6	*p* < 0.0001
How dizzy, sick, or nauseous did you feel during or as a result of the experience?	0.3 (0.2) ^a^	0.4 (0.1) ^a^	0.0 (0.0) ^a^	0.5 (0.2) ^a^	f(3,90) = 1.5	*p* = 0.200
Rate the extent to which you were aware of background sounds in the laboratory where this was actually taking place.	3.2 (0.4) ^a^	2.3 (0.4) ^a^	3.4 (0.4) ^a^	2.2 (0.4) ^a^	f(3,90) = 2.6	*p* = 0.058
Overall, how well do you think that you achieved your task?	5.7 (0.2)	6.0 (0.1) ^a^	5.7 (0.3) ^a^	6.2 (0.1) ^a^	f(3,90) = 1.3	*p* = 0.200

**Table 2:** Participant physiological measurements during treatment. Changes in heart rate and electrodermal activity were significantly higher in the VR treatments. N = 31 for all scenes. Results are Mean (SEM). Results with a different superscript are significantly different (*p* < 0.05).

		LIVESM	HIVESM	LIVEFFR	HIVEFFR	F Ratio	*p*-Value
Baseline Heart Rate (bpm)	Mean	78.0 (2.1)	80.3 (2.3)	78.4 (2.6)	77.8 (2.2)		
Treatment Heart Rate (bpm)	Mean	77.0 (2.3)	90.6 (3.0)	77.4 (2.3)	86.7 (2.2)		
Change in Heart Rate (bpm)	Mean	−1.0 (0.8) ^a^	10.4 (2.0) ^b^	−1.0 (0.8) ^a^	8.9 (1.4) ^b^	f(3,90) = 21.4	<0.0001
Baseline EDA (μmseimans)	Mean	5.8 (0.7)	6.8 (1.1)	5.8 (0.9)	5.7 (1.0)		
Treatment EDA (μmseimans)	Mean	6.3 (0.8)	8.4 (1.2)	6.1 (1.0)	7.2 (1.1)		
Change in EDA (μmseimans)	Mean	0.5 (0.3) ^a^	1.6 (0.4) ^b^	0.3 (0.2) ^a^	1.6 (0.3) ^b^	f(3,90) = 5.10	<0.0023

## Data Availability

The data presented in this study are available on request from the corresponding author. The data are not publicly available due to participants not consenting to having the data publicly available.
